# Marine-Derived Biowaste Conversion into Bioceramic Membrane Materials: Contrasting of Hydroxyapatite Synthesis Methods

**DOI:** 10.3390/molecules26216344

**Published:** 2021-10-20

**Authors:** Yusuf Wibisono, Alien Yala Pratiwi, Christine Ayu Octaviani, Cut Rifda Fadilla, Alfian Noviyanto, Epi Taufik, Muhammad K.H. Uddin, Fajri Anugroho, Nurul Taufiqu Rochman

**Affiliations:** 1 Department of Bioprocess Engineering, Brawijaya University, Jl. Veteran, Malang 65145, Indonesia; alinyalauw@gmail.com (A.Y.P.); christineayuoct@gmail.com (C.A.O.); cutrifda977@gmail.com (C.R.F.); 2 MILI Institute for Water Research, Kawasan Industri Jababeka, Bekasi 17530, Indonesia; 3 Nano Center Indonesia, Jl. PUSPIPTEK Tangerang Selatan, Banten 15314, Indonesia; 4 Department of Mechanical Engineering, Mercu Buana University, Jl. Meruya Selatan, Kebun Jeruk, Jakarta Barat 11650, Indonesia; 5 Faculty of Animal Science, IPB University, Bogor 16680, Indonesia; epitaufik@apps.ipb.ac.id; 6 Department of Science of Dental Materials, Dr. Ishrat-Ul-Ebad Khan Institute of Oral Health Sciences, DOW University of Health Sciences, Karachi 74200, Pakistan; khawaja.hammad@duhs.edu.pk; 7 Department of Environmental Engineering, Brawijaya University, Jl. Veteran, Malang 65145, Indonesia; fajri.anugroho@ub.ac.id; 8 Research Center for Metallurgy and Materials, Indonesian Institute of Sciences, PUSPIPTEK Tangerang Selatan, Banten 15314, Indonesia; nuru006@lipi.go.id

**Keywords:** bioceramic, ultrafiltration membrane, marine biowaste, hydroxyapatite, microwave, coprecipitation, sol–gel, membrane porosity, sintering

## Abstract

Marine-derived biowaste increment is enormous, yet could be converted into valuable biomaterial, e.g., hydroxyapatite-based bioceramic. Bioceramic material possesses superiority in terms of thermal, chemical, and mechanical properties. Bioceramic material also has a high level of biocompatibility when projected into biological tissues. Tuning the porosity of bioceramic material could also provide benefits for bioseparation application, i.e., ultrafiltration ceramic membrane filtration for food and dairy separation processes. This work presents the investigation of hydroxyapatite conversion from crab-shells marine-based biowaste, by comparing three different methods, i.e., microwave, coprecipitation, and sol–gel. The dried crab-shells were milled and calcinated as calcium precursor, then synthesized into hydroxyapatite with the addition of phosphates precursors via microwave, coprecipitation, or sol–gel. The compound and elemental analysis, degree of crystallinity, and particle shape were compared. The chemical compounds and elements from three different methods were similar, yet the degree of crystallinity was different. Higher Ca/P ratio offer benefit in producing a bioceramic ultrafiltration membrane, due to low sintering temperature. The hydroxyapatite from coprecipitation and sol–gel methods showed a significant degree of crystallinity compared with that of the microwave route. However, due to the presence of Fe and Sr impurities, the secondary phase of Ca_9_FeH(PO_4_)_7_ was found in the sol–gel method. The secondary phase compound has high absorbance capacity, an advantage for bioceramic ultrafiltration membranes. Furthermore, the sol–gel method could produce a snake-like shape, compared to the oval shape of the coprecipitation route, another benefit to fabricate porous bioceramic for a membrane filter.

## 1. Introduction

The abundance of marine-derived biowaste or fishery-processing by-products needs to be addressed in search of a sustainable circular economy [1]. From an ecological point of view, the elimination of marine-derived biowaste is in the line with 2030 Sustainable Development Goals Strategies. This conversion into biomaterials could generate new valuable products, invent new technology and create new business [2,3,4]. Hydroxyapatite (HAp) is a valuable biomaterial that could be derived from fishery-processing by-products and marine-derived biowaste [5,6].

Hydroxyapatite is a form of calcium phosphate [Ca_10_(PO_4_)_6_(OH)_2_] that has been extensively utilized as an implant material and has a composition similar to natural hard tissues such as bone and teeth. It has exceptional biocompatibility, osteoconductivity, and osteoinductivity [7,8]. In addition, many studies have shown that HAp ceramics possess no toxicity and no inflammatory effect [9,10]. Therefore, HAp has been extensively used to replace bone tissues, dental implants, tissue engineering, drug carrier, and biosensor applications [11].

Apart from the renowned utilization of HAp in biomedical application [12], tuning HAp into porous materials could be employed for bioceramic membrane filter for separation of gaseous [13,14], aqueous [15,16,17], and biological substances [18,19,20]. When fabricated as bioceramic membrane filters, tailored sintering processes are required to produce specific membrane porosity and tortuosity [21,22,23,24]. The high adsorption capacity of HAp can also be embedded in polymeric materials for wide-range separation [25,26,27,28,29]. The incorporation of HAp particles into polymeric mixed matrix ultrafiltration membranes could enhance permeability and rejection of the membranes [30,31]. Based on those promising results, Hap-based ultrafiltration membranes could also be used to enhance selectivity and isolation of specific compounds, for instance, oligosaccharides from milk [32,33,34]. The application particularly can promote economic benefit in the food industry [35].

HAp can be synthesized in various routes and can be derived from synthetic and natural resources. Synthetic HAp syntheses methods, e.g., precipitation [36], radiofrequency thermal plasma [37], the sol–gel method [38], and hydrothermal methods [39]. However, these methods usually use hazardous chemicals, aging processes, and an imbalanced stoichiometric ratio [40]. Apart from many attempts that have been conducted to use a more environmentally friendly chemical, natural materials have been widely used, e.g., bovine bones [41], fish bones [42], fish scales [43], oyster shells [44], corals [45], eggshells [46], and crab shells [47]. The advantages are twofold—the reduced biowaste and the production of value-added materials.

In this study, we developed HAp from crab shells through microwave, coprecipitation, and sol–gel methods. Producing HAp from crab shells is a low-cost and easy process. Additionally, crab by-products are abundant in Indonesia, since crab is one of Indonesia’s main export commodities. About 25–50% of the total crab bioproduct mass is in the form of crab shells [47]. Crab shells contain around 40–70% calcium carbonate, depending on the species [48]. In many studies, calcium carbonate as a calcium source can be processed further to synthesize hydroxyapatite [49,50,51]. By comparing the results from three different processes—microwave, coprecipitation, and sol–gel methods—this study aimed at investigating the most effective process and economically efficient methods to produce HAp, especially for bioceramic ultrafiltration membranes.

## 2. Results and Discussion

A series of HAp characterization was conducted to examine the results of conversion through microwave, coprecipitation, and sol–gel methods. As described in the Methods Section (Section 3), investigation on the molecular structure, chemical composition, crystallinity, and microstructural morphology were conducted, and the results are presented here.

### 2.1. Molecular Structure

**Figure 1:**
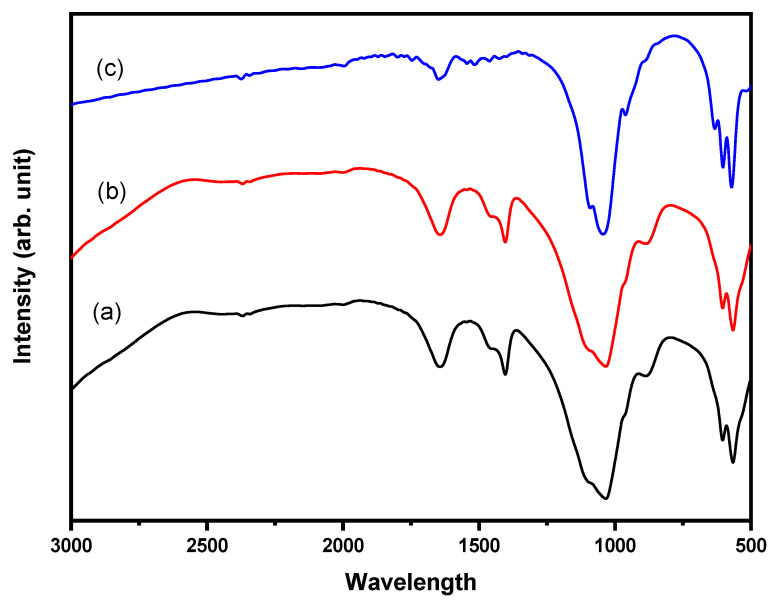
FTIR of HAp synthesized by (**a**) microwave, (**b**) coprecipitation, and (**c**) sol–gel.

Vibrational spectroscopy, i.e., FTIR is susceptible to detect HAp molecular structure, through vibrational frequencies related to HAp chemical bonds [52]. Figure 1 displays the FTIR of HAp synthesize by microwave (Figure 1a), coprecipitation (Figure 1b), and sol–gel (Figure 1c). From FTIR spectra, hydroxyapatite materials have been successfully produced by the presence of PO_4_^3−^ and OH^-^ anions. Phosphate ion absorbance was observed in wave number 963 cm^−1^ with vibration *v1* PO_4_^3−^ ion within wave number 1059 cm^−1^ and 1100 cm^−1^ caused by vibration *v3* PO_4_^3−^ ion. All samples showed their peak at a wavenumber of 1459 cm^−1^, indicating the presence of CO_3_^2−^ anion. The presence of CO_3_^2−^ ion was caused by the interaction between the samples and air during synthesis processes. The CO_3_^2−^ vibration at 1446 cm^−1^ corresponded with aragonite and calcite. At the temperature of 1000 °C, the presence of OH^−^ was higher than that of 600 °C, indicating that the β-tcp phase was mainly produced. The higher temperature could decrease the carbonate groups. 

The FTIR results agreed with the FTIR analysis reported in the literature. Bending vibration of phosphate appeared at 465, 559, and 598 cm^−1^, while the band appeared at 962, 1022, and 1088 cm^−1^, due to stretching vibration [53]. Another report stated that the peaks at 430, 524, 581, 896, 994, 1064, and 1128 cm^−1^, were attributed to the phosphate ions of HAp [54]. Asymmetric stretching vibration *v3* due to the presence of PO_4_^3−^ ion was strongly observed between 1000 and 1150 cm^−^^1^, while asymmetric bending vibration *v4* of PO_4_^3−^ ion was observed between 560 and 610 cm^−1^ [55]. The infrared splitting factor which is calculated by the sum of peak intensities at 604 and 566 cm^−1^ divided by the intensity of the valley between them, can show the degree of crystallinity qualitatively [52]. However, the quantification of crystallinity is better measured by using XRD analysis.

### 2.2. Crystallinity and Chemical Composition

**Figure 2:**
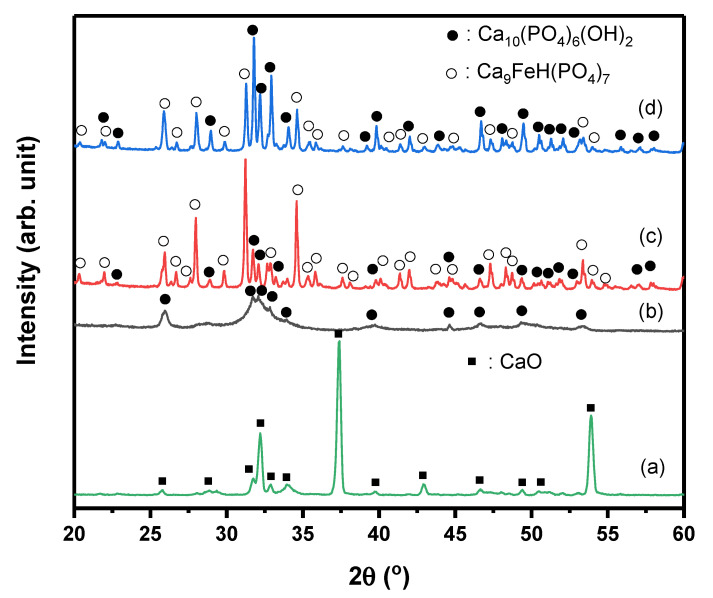
XRD patterns of CaO phase (**a**) and HAp synthesized by (**b**) microwave, (**c**) coprecipitation, and (**d**) sol–gel.

Another important HAp property that was measured is the degree of crystallinity using X-ray diffraction (XRD) measurement. The XRD can determine crystalline structures and quality, crystal size, and lattice defect of the HAp grain [56]. At different treatments, crystal growth of HAp as nanocrystalline materials can be measured by XRD analysis [57]. Figure 2 shows the XRD patterns of HAp synthesized by three different methods, including the synthesized CaO from crab shells (Figure 2). This explains that the reaction of CaO with phosphate-based material to form HAp was successful, showing the formation of HAp phases. Obviously, the final phases depended on the synthesis method, for instance, single-phase HAp, can only be obtained swiftly and evenly by synthesizing using the microwave, as shown in Figure 2b. This result agrees with previous reports in which HAp was successfully synthesized by microwave [58,59,60]. The typical XRD patterns are similar to those reports, whereby low crystallinity was observed in XRD patterns. Indeed, the crystallinity of HAp could be enhanced by calcination at high temperatures, i.e., >1000 °C [61]; however, the particle size is more likely to grow during calcination.

As shown in Figure 2b, the diffractogram peaks were observed at angles 2θ 25.942°, 31.719°, 32.152°, and 32.877°, similar to the standard of JCPDS [62]. However, the crystallinity of microwave HAp was lower, about 25%, compared with the HAp coprecipitation (Figure 2c) and HAp sol–gel (Figure 2d), which were approximately 84% and 92%, respectively.

Nevertheless, the coprecipitation and sol–gel method showed the presence of Ca_9_FeH(PO_4_)_7_ phase, in addition to the HAp phase. Interestingly, the Ca_9_FeH(PO_4_)_7_ phase was dominant in the coprecipitation method, showing the high-intensity peak at 31.2°, even though the calcination temperature was similar to the sol–gel method, i.e., 1000 °C. To verify the presence of Fe impurities, the XRF measurement was conducted.

According to Table 1, the amount of Fe for the three methods was similar, about 1.1 wt.%, while the amount of Ca for microwave, coprecipitation, and sol–gel was 74.35, 78.02, and 79.91 wt.%, respectively. The difference in the Ca content is more likely due to the synthesis method, whereby coprecipitation and sol–gel could maintain the amount higher than 78%. To confirm this result, HAp was synthesized using a microwave at 600 W, and the result showed the amount of Ca and P was 75.94 and 19.6 wt.%, respectively. This indicates that the power of the microwave influences the number of elements. Moreover, the Ca/P ratio of the microwave was the lowest, compared with other methods, i.e., 2.69, as shown in Table 1, while the other method showing the Ca/P ratio > 4. This is the one possible reason that microwave synthesis produces a single-phase HAp. The fabrication of bioceramic ultrafiltration membranes could benefit from the high Ca/P ratio; the details are discussed in Section 2.4 as an outlook.

However, the microwave method prevented the reaction between Fe and HAp to form Ca_9_FeH(PO_4_)_7_. It seems the power of microwave used in this study was not enough to induce reaction between Fe and HAp, as characterized by lower crystallinity in Figure 2b compared with Figure 2c,d. As shown in Figure 2b, the main peak of HAp synthesized by microwave was very broad, significantly different from HAp synthesized by coprecipitation (Figure 2c) and sol–gel (Figure 2d). Broadening of the XRD peak indicates that crystallite size decreased. The crystallite size was estimated from peak 31.719° (121). Indeed, the crystallite size of HAp synthesized by microwave was very fine, i.e., 10.3 nm, compared with those coprecipitation and sol–gel, as shown in Table 2.

Nevertheless, the broadening of the peak in the XRD of microwave HAp was also influenced by the strain. The crystallite sizes of coprecipitation and sol–gel methods, which were 179 and 96.7 nm, respectively, increased more than nine times, compared with the microwave method.

### 2.3. HAp Microstructure

**Figure 3:**
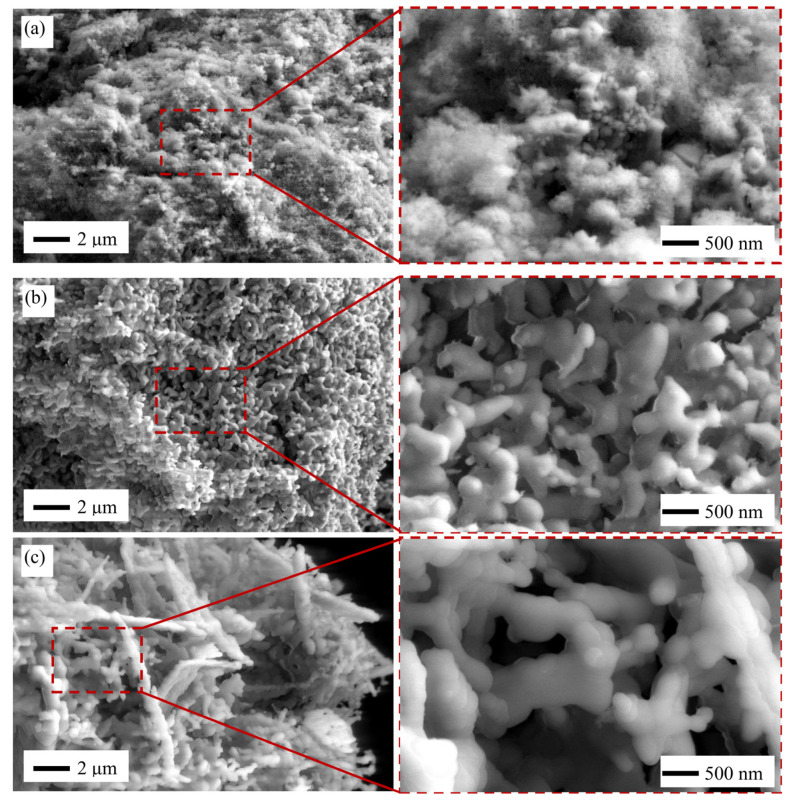
SEM images of HAp synthesized by (**a**) microwave, (**b**) coprecipitation, and (**c**) sol–gel with its magnification.

Figure 3 presents the SEM images of HAp microstructure obtained by three different synthesis methods. Indeed, the HAp synthesized by microwave showed fine microstructure, as shown in Figure 3a and its magnification. The average particle size of microwave HAp was 100 ± 29 nm.

As shown in the magnification of Figure 3a, there are a lot of very fine microstructure microwave HAp which cannot be clearly seen. The planetary or spherical shapes with fewer pores were observed similar to the finding from Lamkhao et al. [63]. It seems that these were HAp particles, and the size was about 10–30 nm, which is almost similar to the crystallite size. This result also confirmed the low crystallinity of microwave HAp, as shown in Figure 2b. However, the microwave HAp synthesis method has several advantages, e.g., rapid heating, less reaction time, and more energy efficiency [64].

In contrast, the microstructure of coprecipitation (Figure 3b) and sol–gel (Figure 3c) experienced grain growth noticeably, compared with microwave HAp. The average particle size of coprecipitation and sol–gel HAp was 465 ± 107 and 522 ± 206 nm, respectively. Although the calcination temperature was similar for the coprecipitation and sol–gel HAp, the size and shape were quite different. Coprecipitation showed an irregular shape, which tended to form an oval shape, as shown in Figure 3b. According to Shojai et al. [65], HAp synthesis using coprecipitation methods could produce several crystalline shapes such as irregular, sphere, rod/needle-like, plate, etc.

Furthermore, HAp synthesized by the sol–gel method showed a snake-like shape, as shown in Figure 3c. This snake-like shape is reported by Noviyanto et al. [66] while synthesizing nano-sized La_2_Ti_2_O_7_ using chemothermal pulverization. The sol–gel method could produce HAp shapes such as irregular, sphere, and rod/needle tube [65]. Agitation speed, pH, and temperature during HAp synthesis affect the grain size and shape [67].

### 2.4. An Outlook: Bioceramic Ultrafiltration Membrane Application

When utilized as a biomaterial, HAp must possess a high level of biocompatibility [68], which has a Ca/P ratio is 1.67. However, the purpose of this study was to evaluate the potential use of the HAp as a bioceramic ultrafiltration membrane. The HAp produced by all methods in this study had a higher Ca/P ratio, compared with stoichiometry HAp because it was extracted from natural resources of marine-derived biowaste, i.e., crab shells, resulting in the presence of trace elements responsible for the non-stoichiometric HAp, including the existence of CaO in the HAp produced [69].

As the basic material for bioceramic ultrafiltration membranes, CaO has many advantages. Sintered pure CaO has many micropores, and the pore size could be enhanced by adding some additives [70]. During the sintering process, the dense and crystallized CaO show a low sintering temperature, i.e., below 900 °C, and low dielectric constant, and it has higher flexural strength above 134 MPa [71]. These properties imply that the material is appropriate for bioceramic ultrafiltration membranes. When sintered at a higher temperature such as 1300 °C for 3 h, the pure CaO exhibit the best densification [72]. Tuning the bioceramic membrane pores by using additives or pore-forming agents could be added in the CaO sintering processes [73,74,75].

The existence of calcium iron hydrogen phosphate or Ca_9_FeH(PO_4_)_7_ is also another advantage, although its benefit for bioceramic ultrafiltration membranes is still unknown. The compound has a high capability as an absorber from, e.g., in UV light, as cosmeceutical, or used in water or wastewater treatment [76,77,78,79].

## 3. Materials and Methods

### 3.1. Materials

**Figure 4:**
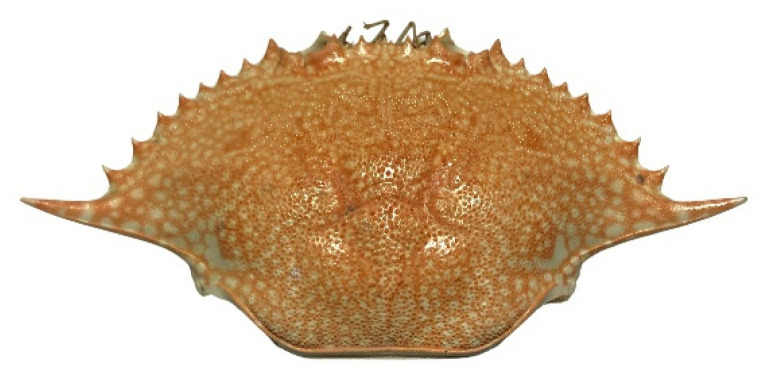
The dried crab-shell as calcium precursor.

The crab-shell (*Portunus pelagicus*) biowaste as calcium precursor (Figure 4) was obtained from the marine industry in the north coastal line of Pasuruan, East Java, Indonesia. Some chemicals were used in this research, e.g., diammonium phosphate (NH_4_)_2_HPO_4_, hydrochloric acid HCl 37%, phosphoric acid H_3_PO_4_ 85%, ammonia NH_3_ 25%, and ethanol 96% are purchased from Merck KGaA (Darmstadt, Germany).

### 3.2. Preparation of Calcium Oxide

The crab shells were washed to remove remained crab meat and other impurities. The crab shells then dried under open sun drying for 12 h, followed by 4 h of drying in the electric oven until reaching 9.5 ± 0.12% wet basis moisture content. The dried crab shells were milled into a fine powder using a pilot-size tubular ball mill (rotation speed 84.6 rpm, 1:10 crab shells, and stainless-steel balls ratio) for 4 h [80]. The produced powder was then sieved using woven wire 325 mesh sieve and produced crab-shell powder with 44 µm in sizes. The sieved powder was further milled with a custom-made Planetary Ball Mill (double 500 mL stainless steel vials, rotation speed 450 rpm) and produced powder with 4.6 µm particle sizes. The obtained powder sample was dissolved in Tween-80 polysorbate surfactant solution and characterized using a particle size analyzer (Malvern Nano–ZS, UK). The powder was then calcinated at a temperature of 1000 °C for 5 h and produced calcium oxide (CaO) powder. The CaO powders were then synthesized into Hap materials by using three different methods, i.e., microwave, coprecipitation, and sol–gel methods, adapted and modified from [65].

### 3.3. Synthesis of Hydroxyapatite: Microwave

A total of 6.25 g CaO was dissolved in 100 mL HCl 37% 1:3 in beaker glass; then, 262.5 mL of (NH4)_2_HPO_4_ 0.25 M was added to the mixture. The mixture was stirred vigorously for 30 min using a magnetic stirrer (Thermo scientific Cimarec+, Waltham, MA, USA). The pH of the mixture was adjusted to 10–11 by adding 1:1 25% liquor NH_4_OH and demineralized water. The mixture was then immediately heated using the microwave at the power of 600 Watt and 800 Watt for 15 min. The precipitate formed was filtered using filter paper with a pore size of approximately 8–11 µm and washed with demineralized water to eliminate the remaining ammonia. It was then dried using an oven (Binder RedLine, Tuttlingen, Germany) at 100 °C for 24 h. Dried HAp precipitate was ground to produce fine powder using mortar and pestle. The step was repeated several times by varying the particle sizes and microwave power [81]. The synthesis condition is summarized in Table 3.

### 3.4. Synthesis of Hydroxyapatite: Coprecipitation

The coprecipitation method was based on the previous procedure with slight modification [24]. The amount of 22 g of CaO powder was dissolved in demineralized water and mixed with diluted H_3_PO_4_ 85% (60 °C, agitation speed 700 rpm). The H_3_PO_4_ was added by titration process with a drop rate of 3 mL/minute. NH_3_ was then added to the suspension until it reached pH 10. Subsequently, the suspension was then sonicated with an ultrasonic bath (Branson 3510, Brookfield, CT, USA) for 1 h at 42 kHz. The suspension was placed at room temperature for 24 h. The obtained sediment was washed with demineralized water and filtered using filter paper with a pore size of approximately 8–11 µm. The sediment was dried in an oven (Binder RedLine, Tuttlingen, Germany) for 15 h at 90 °C and calcinated at 1000 °C for 6 h. The synthesis condition is summarized in Table 3.

### 3.5. Synthesis of Hydroxyapatite: Sol-Gel

The amount of 7.4 g of CaO powder was dissolved in 100 mL of ethanol 96% and mixed with 100 mL of H_3_PO_4_ that has been diluted in demineralized water (temperature of 37 °C, agitation speed of 300 rpm). The H_3_PO_4_ was added by titration process with a drop rate of 1 mL/minute. The NH_3_ was added to the suspension until it has reached a pH of 10. The suspension was placed at room temperature for 24 h. The obtained gelatin was washed with demineralized water 3 times and then filtered using filter paper with a pore size of approximately 8–11 µm. The sediment was then dried in an oven (Binder RedLine, Tuttlingen, Germany) for 18 h at 105 °C and subsequently calcinated at 1000 °C for 6 h [82]. The synthesis condition is summarized in Table 3.

### 3.6. Characterization of Hydroxyapatite

The HAp powder was then characterized using a Fourier transform-infrared (Shimadzu 8400S, Kyoto, Japan), X-ray diffraction (PANalytical X’Pert3 Powder, Malvern, UK), X-ray fluorescence (PANalytical Minipal 4, Malvern, UK), and scanning electron microscope (FEI Quanta FEG 650, Hillsboro, OR, USA).

## 4. Conclusions

Hydroxyapatite-based bioceramic particles derived from crab shells biowaste were successfully synthesized by using three different methods—microwave, coprecipitation, and sol–gel. Analysis of chemical compounds by FTIR showed that the functional groups from HAp produced from three different routes were similar. Based on the XRD results, the degree of crystallinity of HAp synthesized by using the microwave method was much lower than the other two methods. Elemental analysis using XRF showed that Fe and Sr elements existed and acted as impurities, which lead to the formation of Ca_9_FeH(PO_4_)_7_ that was observed in the sol–gel method. CaO as the main constituent offers the best properties as raw material for bioceramic ultrafiltration membranes.

## Figures and Tables

**Table 1:** Elemental analysis of HAp synthesized by microwave, coprecipitation, and sol–gel.

Methods	Elements (wt.%)									
	Ca	P	Ti	Mn	Fe	Cu	Sr	Zr	Others	Ca/P
Microwave	74.35	21.3	0.074	0.27	1.09	0.066	2.3	0.3	0.250	2.69
Coprecipitation	78.02	17.3	0.063	0.28	1.10	0.068	2.5	0.3	0.369	2.73
Sol–Gel	79.34	16.4	0.068	0.26	1.08	0.065	2.2	0.3	0.287	4.84

**Table 2:** Crystallite size, strain, and particle size of HAp synthesized by microwave, coprecipitation, and sol–gel.

Methods	Crystallite Size (nm)	Strain (%)	Particle Size (nm)
Microwave	10.3	0.98	100 ± 29
Coprecipitation	179	0.09	465 ± 107
Sol–Gel	96.7	0.11	522 ± 206

**Table 3:** Hydroxyapatite conversion by microwave, coprecipitation, and sol–gel methods.

Methods	Thermal-Induced Reaction	Time (min)
Microwave	600 W; 800 W	15
Coprecipitation	1000 °C	360
Sol–Gel	1000 °C	360
